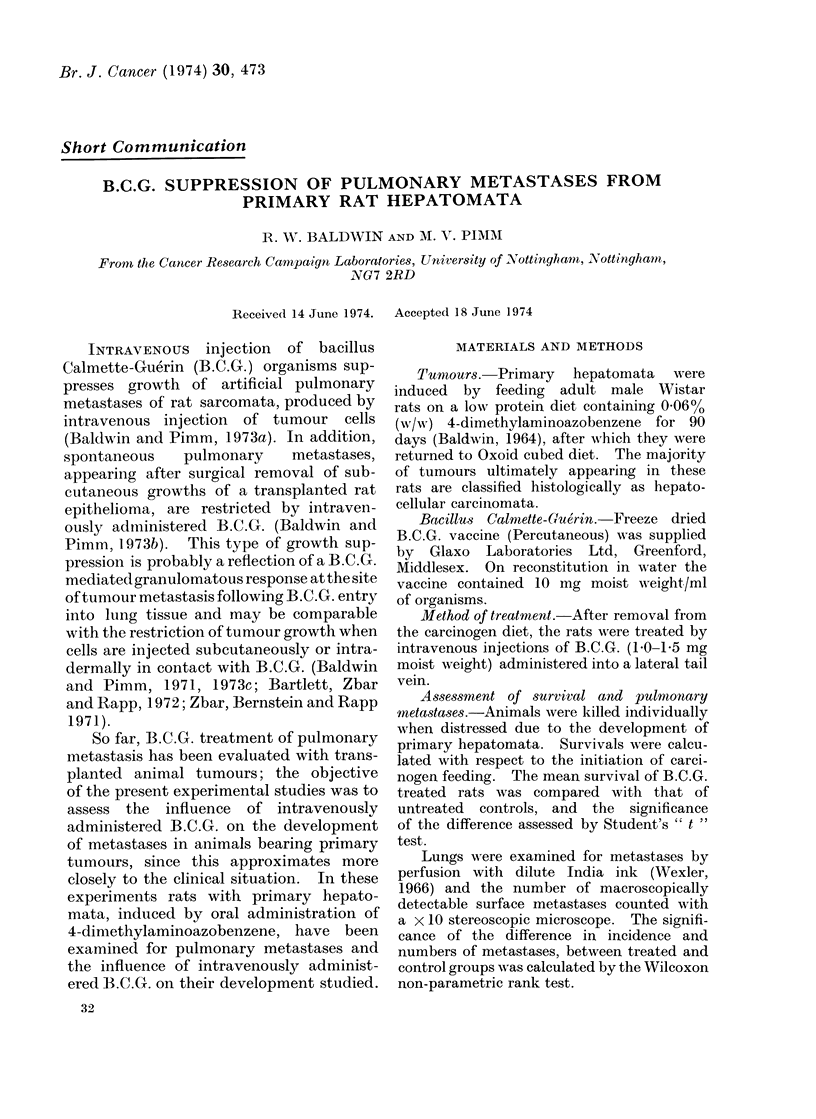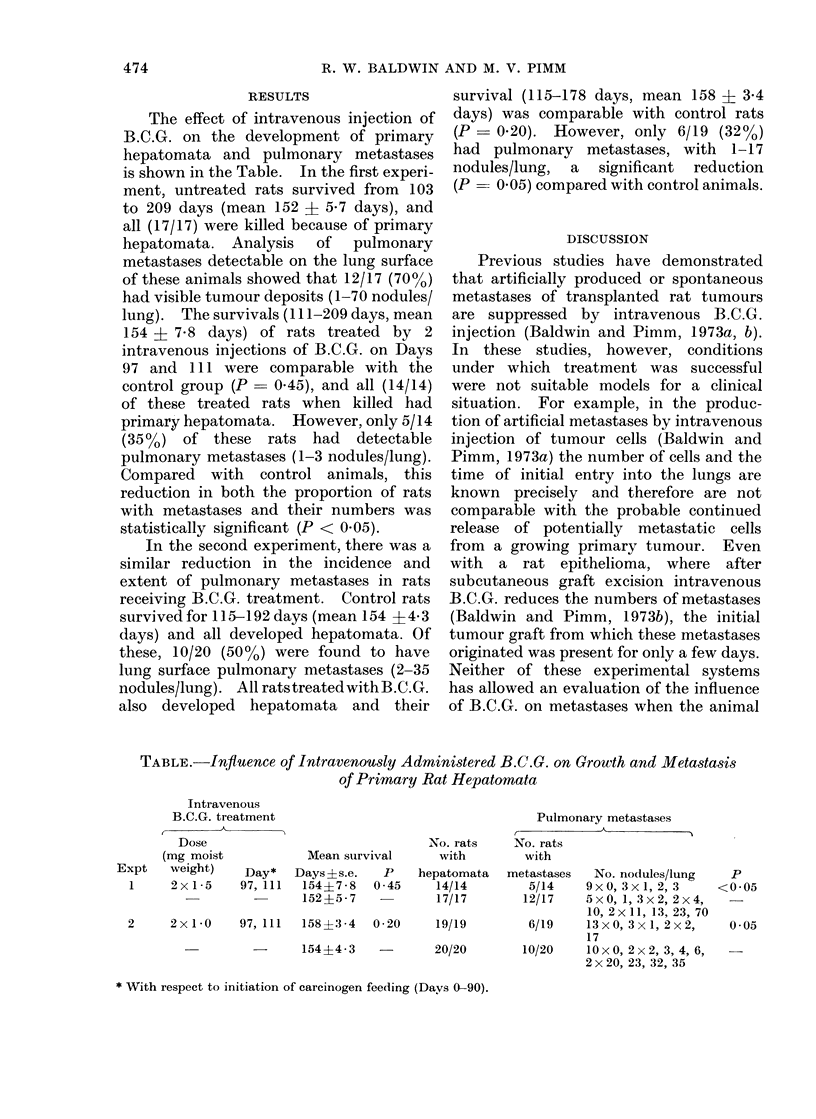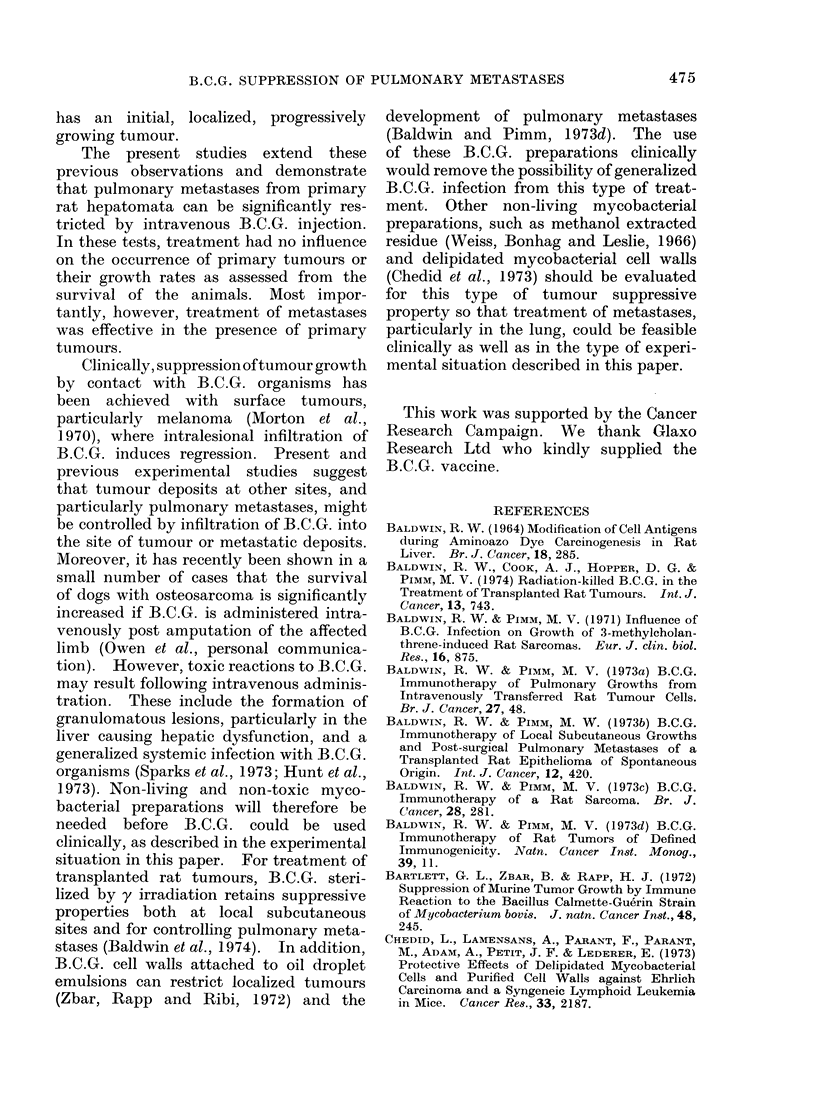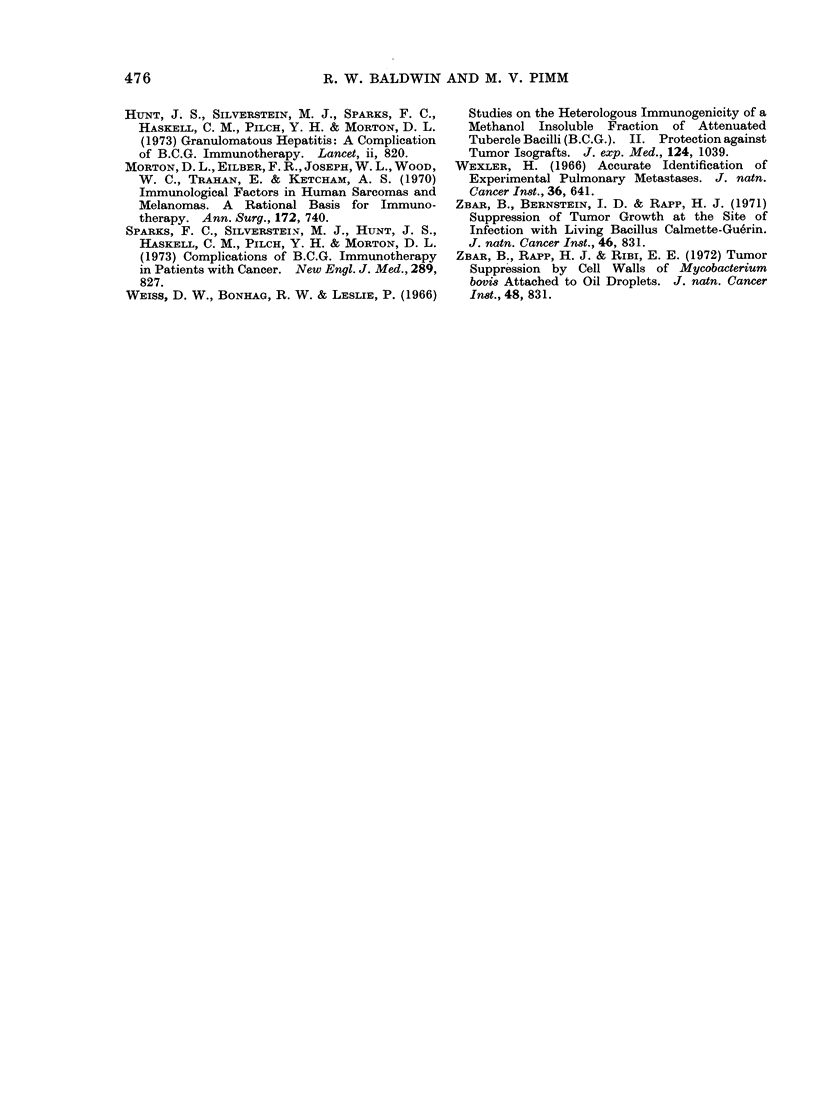# B.C.G. suppression of pulmonary metastases from primary rat hepatomata.

**DOI:** 10.1038/bjc.1974.223

**Published:** 1974-11

**Authors:** R. W. Baldwin, M. V. Pimm


Br. J. Cancer (1974) 30, 473

### Short Communication

### B.C.G. SUPPRESSION OF PULMONARY METASTASES FROM PRIMARY RAT HEPATOMATA

R. W. BALDWIN AND M. V. PIMM

*From the Cancer Research Campaign Laboratories, University of Nottingham, Nottingham, NG7 2RD*

Received 14 June 1974. Accepted 18 June 1974

INTRAVENOUS injection of bacillus Calmette-Guérin (B.C.G.) organisms suppresses growth of artificial pulmonary metastases of rat sarcomata, produced by intravenous injection of tumour cells (Baldwin and Pimm, 1973*a*). In addition, spontaneous pulmonary metastases, appearing after surgical removal of subcutaneous growths of a transplanted rat epithelioma, are restricted by intravenously administered B.C.G. (Baldwin and Pimm, 1973*b*). This type of growth suppression is probably a reflection of a B.C.G. mediated granulomatous response at the site of tumour metastasis following B.C.G. entry into lung tissue and may be comparable with the restriction of tumour growth when cells are injected subcutaneously or intradermally in contact with B.C.G. (Baldwin and Pimm, 1971, 1973*c*; Bartlett, Zbar and Rapp, 1972; Zbar, Bernstein and Rapp 1971).

So far, B.C.G. treatment of pulmonary metastasis has been evaluated with transplanted animal tumours; the objective of the present experimental studies was to assess the influence of intravenously administered B.C.G. on the development of metastases in animals bearing primary tumours, since this approximates more closely to the clinical situation. In these experiments rats with primary hepatomata, induced by oral administration of 4-dimethylaminoazobenzene, have been examined for pulmonary metastases and the influence of intravenously administered B.C.G. on their development studied.

### MATERIALS AND METHODS

*Tumours.*—Primary hepatomata were induced by feeding adult male Wistar rats on a low protein diet containing 0·06% (w/w) 4-dimethylaminoazobenzene for 90 days (Baldwin, 1964), after which they were returned to Oxoid cubed diet. The majority of tumours ultimately appearing in these rats are classified histologically as hepatocellular carcinomata.

*Bacillus Calmette-Guérin.*—Freeze dried B.C.G. vaccine (Percutaneous) was supplied by Glaxo Laboratories Ltd, Greenford, Middlesex. On reconstitution in water the vaccine contained 10 mg moist weight/ml of organisms.

*Method of treatment.*—After removal from the carcinogen diet, the rats were treated by intravenous injections of B.C.G. (1·0–1·5 mg moist weight) administered into a lateral tail vein.

*Assessment of survival and pulmonary metastases.*—Animals were killed individually when distressed due to the development of primary hepatomata. Survivals were calculated with respect to the initiation of carcinogen feeding. The mean survival of B.C.G. treated rats was compared with that of untreated controls, and the significance of the difference assessed by Student's "$t$" test.

Lungs were examined for metastases by perfusion with dilute India ink (Wexler, 1966) and the number of macroscopically detectable surface metastases counted with a $\times 10$ stereoscopic microscope. The significance of the difference in incidence and numbers of metastases, between treated and control groups was calculated by the Wilcoxon non-parametric rank test.

### RESULTS

The effect of intravenous injection of B.C.G. on the development of primary hepatomata and pulmonary metastases is shown in the Table. In the first experiment, untreated rats survived from 103 to 209 days (mean 152 ± 5·7 days), and all (17/17) were killed because of primary hepatomata. Analysis of pulmonary metastases detectable on the lung surface of these animals showed that 12/17 (70%) had visible tumour deposits (1–70 nodules/lung). The survivals (111–209 days, mean 154 ± 7·8 days) of rats treated by 2 intravenous injections of B.C.G. on Days 97 and 111 were comparable with the control group ($P = 0{\cdot}45$), and all (14/14) of these treated rats when killed had primary hepatomata. However, only 5/14 (35%) of these rats had detectable pulmonary metastases (1–3 nodules/lung). Compared with control animals, this reduction in both the proportion of rats with metastases and their numbers was statistically significant ($P < 0{\cdot}05$).

In the second experiment, there was a similar reduction in the incidence and extent of pulmonary metastases in rats receiving B.C.G. treatment. Control rats survived for 115–192 days (mean 154 ±4·3 days) and all developed hepatomata. Of these, 10/20 (50%) were found to have lung surface pulmonary metastases (2–35 nodules/lung). All rats treated with B.C.G. also developed hepatomata and their survival (115–178 days, mean 158 ± 3·4 days) was comparable with control rats ($P = 0{\cdot}20$). However, only 6/19 (32%) had pulmonary metastases, with 1–17 nodules/lung, a significant reduction ($P = 0{\cdot}05$) compared with control animals.

### DISCUSSION

Previous studies have demonstrated that artificially produced or spontaneous metastases of transplanted rat tumours are suppressed by intravenous B.C.G. injection (Baldwin and Pimm, 1973*a*, *b*). In these studies, however, conditions under which treatment was successful were not suitable models for a clinical situation. For example, in the production of artificial metastases by intravenous injection of tumour cells (Baldwin and Pimm, 1973*a*) the number of cells and the time of initial entry into the lungs are known precisely and therefore are not comparable with the probable continued release of potentially metastatic cells from a growing primary tumour. Even with a rat epithelioma, where after subcutaneous graft excision intravenous B.C.G. reduces the numbers of metastases (Baldwin and Pimm, 1973*b*), the initial tumour graft from which these metastases originated was present for only a few days. Neither of these experimental systems has allowed an evaluation of the influence of B.C.G. on metastases when the animal

TABLE.—*Influence of Intravenously Administered B.C.G. on Growth and Metastasis of Primary Rat Hepatomata*

| Expt | Intravenous B.C.G. treatment: Dose (mg moist weight) | Day* | Mean survival: Days ± s.e. | Mean survival: *P* | No. rats with hepatomata | Pulmonary metastases: No. rats with metastases | Pulmonary metastases: No. nodules/lung | *P* |
|---|---|---|---|---|---|---|---|---|
| 1 | 2 × 1·5 | 97, 111 | 154 ± 7·8 | 0·45 | 14/14 | 5/14 | 9 × 0, 3 × 1, 2, 3 | < 0·05 |
| | — | — | 152 ± 5·7 | — | 17/17 | 12/17 | 5 × 0, 1, 3 × 2, 2 × 4, 10, 2 × 11, 13, 23, 70 | — |
| 2 | 2 × 1·0 | 97, 111 | 158 ± 3·4 | 0·20 | 19/19 | 6/19 | 13 × 0, 3 × 1, 2 × 2, 17 | 0·05 |
| | — | — | 154 ± 4·3 | — | 20/20 | 10/20 | 10 × 0, 2 × 2, 3, 4, 6, 2 × 20, 23, 32, 35 | — |

* With respect to initiation of carcinogen feeding (Days 0–90).

has an initial, localized, progressively growing tumour.

The present studies extend these previous observations and demonstrate that pulmonary metastases from primary rat hepatomata can be significantly restricted by intravenous B.C.G. injection. In these tests, treatment had no influence on the occurrence of primary tumours or their growth rates as assessed from the survival of the animals. Most importantly, however, treatment of metastases was effective in the presence of primary tumours.

Clinically, suppression of tumour growth by contact with B.C.G. organisms has been achieved with surface tumours, particularly melanoma (Morton *et al.*, 1970), where intralesional infiltration of B.C.G. induces regression. Present and previous experimental studies suggest that tumour deposits at other sites, and particularly pulmonary metastases, might be controlled by infiltration of B.C.G. into the site of tumour or metastatic deposits. Moreover, it has recently been shown in a small number of cases that the survival of dogs with osteosarcoma is significantly increased if B.C.G. is administered intravenously post amputation of the affected limb (Owen *et al.*, personal communication). However, toxic reactions to B.C.G. may result following intravenous administration. These include the formation of granulomatous lesions, particularly in the liver causing hepatic dysfunction, and a generalized systemic infection with B.C.G. organisms (Sparks *et al.*, 1973; Hunt *et al.*, 1973). Non-living and non-toxic mycobacterial preparations will therefore be needed before B.C.G. could be used clinically, as described in the experimental situation in this paper. For treatment of transplanted rat tumours, B.C.G. sterilized by $\gamma$ irradiation retains suppressive properties both at local subcutaneous sites and for controlling pulmonary metastases (Baldwin *et al.*, 1974). In addition, B.C.G. cell walls attached to oil droplet emulsions can restrict localized tumours (Zbar, Rapp and Ribi, 1972) and the development of pulmonary metastases (Baldwin and Pimm, 1973*d*). The use of these B.C.G. preparations clinically would remove the possibility of generalized B.C.G. infection from this type of treatment. Other non-living mycobacterial preparations, such as methanol extracted residue (Weiss, Bonhag and Leslie, 1966) and delipidated mycobacterial cell walls (Chedid *et al.*, 1973) should be evaluated for this type of tumour suppressive property so that treatment of metastases, particularly in the lung, could be feasible clinically as well as in the type of experimental situation described in this paper.

This work was supported by the Cancer Research Campaign. We thank Glaxo Research Ltd who kindly supplied the B.C.G. vaccine.

### REFERENCES

Baldwin, R. W. (1964) Modification of Cell Antigens during Aminoazo Dye Carcinogenesis in Rat Liver. *Br. J. Cancer*, **18**, 285.

Baldwin, R. W., Cook, A. J., Hopper, D. G. & Pimm, M. V. (1974) Radiation-killed B.C.G. in the Treatment of Transplanted Rat Tumours. *Int. J. Cancer*, **13**, 743.

Baldwin, R. W. & Pimm, M. V. (1971) Influence of B.C.G. Infection on Growth of 3-methylcholanthrene-induced Rat Sarcomas. *Eur. J. clin. biol. Res.*, **16**, 875.

Baldwin, R. W. & Pimm, M. V. (1973*a*) B.C.G. Immunotherapy of Pulmonary Growths from Intravenously Transferred Rat Tumour Cells. *Br. J. Cancer*, **27**, 48.

Baldwin, R. W. & Pimm, M. W. (1973*b*) B.C.G. Immunotherapy of Local Subcutaneous Growths and Post-surgical Pulmonary Metastases of a Transplanted Rat Epithelioma of Spontaneous Origin. *Int. J. Cancer*, **12**, 420.

Baldwin, R. W. & Pimm, M. V. (1973*c*) B.C.G. Immunotherapy of a Rat Sarcoma. *Br. J. Cancer*, **28**, 281.

Baldwin, R. W. & Pimm, M. V. (1973*d*) B.C.G. Immunotherapy of Rat Tumors of Defined Immunogenicity. *Natn. Cancer Inst. Monog.*, **39**, 11.

Bartlett, G. L., Zbar, B. & Rapp, H. J. (1972) Suppression of Murine Tumor Growth by Immune Reaction to the Bacillus Calmette-Guérin Strain of *Mycobacterium bovis*. *J. natn. Cancer Inst.*, **48**, 245.

Chedid, L., Lamensans, A., Parant, F., Parant, M., Adam, A., Petit, J. F. & Lederer, E. (1973) Protective Effects of Delipidated Mycobacterial Cells and Purified Cell Walls against Ehrlich Carcinoma and a Syngeneic Lymphoid Leukemia in Mice. *Cancer Res.*, **33**, 2187.

HUNT, J. S., SILVERSTEIN, M. J., SPARKS, F. C., HASKELL, C. M., PILCH, Y. H. & MORTON, D. L. (1973) Granulomatous Hepatitis: A Complication of B.C.G. Immunotherapy. *Lancet*, ii, 820.

MORTON, D. L., EILBER, F. R., JOSEPH, W. L., WOOD, W. C., TRAHAN, E. & KETCHAM, A. S. (1970) Immunological Factors in Human Sarcomas and Melanomas. A Rational Basis for Immunotherapy. *Ann. Surg.*, **172**, 740.

SPARKS, F. C., SILVERSTEIN, M. J., HUNT, J. S., HASKELL, C. M., PILCH, Y. H. & MORTON, D. L. (1973) Complications of B.C.G. Immunotherapy in Patients with Cancer. *New Engl. J. Med.*, **289**, 827.

WEISS, D. W., BONHAG, R. W. & LESLIE, P. (1966) Studies on the Heterologous Immunogenicity of a Methanol Insoluble Fraction of Attenuated Tubercle Bacilli (B.C.G.). II. Protection against Tumor Isografts. *J. exp. Med.*, **124**, 1039.

WEXLER, H. (1966) Accurate Identification of Experimental Pulmonary Metastases. *J. natn. Cancer Inst.*, **36**, 641.

ZBAR, B., BERNSTEIN, I. D. & RAPP, H. J. (1971) Suppression of Tumor Growth at the Site of Infection with Living Bacillus Calmette-Guérin. *J. natn. Cancer Inst.*, **46**, 831.

ZBAR, B., RAPP, H. J. & RIBI, E. E. (1972) Tumor Suppression by Cell Walls of *Mycobacterium bovis* Attached to Oil Droplets. *J. natn. Cancer Inst.*, **48**, 831.